# P-264. Beyond Screening: Leveraging Electronic Health Record Data to Identify Out-of-Care People Living With HIV in the Emergency Department

**DOI:** 10.1093/ofid/ofaf695.485

**Published:** 2026-01-11

**Authors:** Douglas A E White, Jacob Manteuffel, Molly Burns, Montana Jewett, Cedric Rodriguez, Gabriela Regalado, Cinthya Mujica Pinto, Kevin Del Angel, Christian Loszewski, Dalton Bender, Jo-Ann Rammal, Shannon Payne, Sharon Zahul, William Grace, Indira Brar, Joseph Miller, Mit Patel, Bijou R Hunt

**Affiliations:** Alameda Health System, Oakland, CA; Henry Ford Hospital, Detroit, Michigan; Alameda Health System, Oakland, CA; Alameda Health System, Oakland, CA; Alameda Health System, Oakland, CA; Alameda Health System, Oakland, CA; Alameda Health System, Oakland, CA; Alameda Health Associate, Oakland, California; Henry Ford Health, Detroit, Michigan; Henry Ford Health, Detroit, Michigan; Henry Ford Health, Detroit, Michigan; Henry Ford Health, Detroit, Michigan; Henry Ford Health, Detroit, Michigan; Henry Ford, Detroit, Michigan; Henry Ford Hospital, Detroit, Michigan; Henry Ford Health, Detroit, Michigan; Henry Ford Health, Detroit, Michigan; Sinai Infectious Disease Center, Chicago, Illinois

## Abstract

**Background:**

Emergency department (ED) HIV screening programs aim to identify new HIV cases but often uncover individuals previously diagnosed with HIV (PLWH Dx), many of whom are out of care (OOC) and require re-engagement. This highlights a potential missed opportunity—as many more OOC PLWH Dx likely go undetected if identification relies solely on repeat testing via screening programs. Re-engaging these individuals to care is vital to Ending the HIV Epidemic (EHE) efforts, as they often have unsuppressed viral loads and account for a majority of HIV transmissions in the US. Given the high ED utilization by PLWH, the ED represents a strategic setting for both their identification and re-engagement in care. This study compared the identification of OOC PLWH Dx using an electronic health record algorithm (EHRa) versus an ED HIV screening program.

**Methods:**

This 6-month retrospective cross-sectional study was conducted at two EDs with existing HIV screening programs. Each site developed an EHRa using historical lab data and keyword-based medical history to identify ED patients as a PLWH. EHRa-identified PLWH were reviewed by infectious disease or research staff and classified as in care (IC) or OOC using site-specific criteria via chart review, patient interview, and state health department data. HIV screening data were obtained from site-level databases maintained by HIV navigators.

**Results:**

The EHRa identified 763 PLWH: 590 (77.3%) IC and 173 (22.7%) OOC. Mean age was 47; 69% male, 11% White, 73% Black, 14% Hispanic, and 9% non-English-speaking. During the same period, 14,212 HIV screening identified 42 (0.3%) new diagnoses and 136 (1.0%) PLWH Dx: 84 (61.8%) IC and 52 (38.2%) OOC. All 136 PLWH Dx via repeat testing were also identified by the EHRa. The EHRa identified an additional 121 OOC PLWH Dx missed by screening.

**Conclusion:**

An EHR-based algorithm identified significantly more OOC PLWH Dx than screening alone. These findings suggest that relying solely on ED screening may miss key opportunities to re-engage PLWH. Integrating EHR tools into ED workflows may enhance EHE efforts by enabling targeted re-linkage to care.

**Disclosures:**

Douglas AE White, MD, Gilead Sciences: Grant/Research Support Jacob Manteuffel, MD, Cepheid: Honoraria|Gilead Sciences: Grant/Research Support|Indivior: Advisor/Consultant Indira Brar, MD, Gilead: Advisor/Consultant|Gilead: Grant/Research Support|Gilead: Honoraria|ViiV Healthcare: Advisor/Consultant|ViiV Healthcare: Grant/Research Support|ViiV Healthcare: Honoraria Joseph Miller, MD, Astrazeneca: Advisor/Consultant Bijou R. Hunt, MA, Gilead Sciences: Grant/Research Support

